# Tomotherapy With Synchrony Fiducial Tracking for Stereotactic Body Radiotherapy in Prostate Cancer: A Single-Center Experience on Toxicity

**DOI:** 10.7759/cureus.85083

**Published:** 2025-05-30

**Authors:** Lorella Lo Conte, Vincenzo Iorio, Ida Rosalia Scognamiglio, Antonio Russo, Ersilia Donda, Alfonsina Pepe, Anna Viggiano, Ferdinando Francomacaro, Ivona Zlatkova, Caterina Muto

**Affiliations:** 1 Radiotherapy, Emicenter, Naples, ITA

**Keywords:** prostate cancer, sbrt, stereotactic body radiation therapy, synchrony fiducial tracking, tomotherapy, toxicity

## Abstract

The use of stereotactic body radiation therapy (SBRT) in the treatment of prostate cancer (PC) has increased significantly in recent years. This study aims to evaluate the safety of patients with localized PC undergoing SBRT using Radixact Tomotherapy with Synchrony fiducial tracking, a real-time motion tracking and correction system. This retrospective work analyzes 43 PC patients treated with SBRT from June 2022 to February 2025. Among these, nine (21%) were classified as low risk, 15 (35%) as favorable intermediate risk, 13 (30%) as unfavorable intermediate risk, and six (14%) as high risk. Androgen deprivation therapy (ADT) was prescribed based on the risk group. Three gold fiducial markers were placed in the prostate under ultrasound guidance before a computed tomography (CT) scan to visualize the position and movement of the prostate before and during treatment, allowing for real-time corrections. The total dose administered was 36.25 Gy in five fractions, delivered every other day. The required target coverage was at least 95% of the planning target volume (PTV) with at least 95% of the prescribed dose. The Common Terminology Criteria for Adverse Events (CTCAE) version 5.0 was used to evaluate acute and late toxicity, including gastrointestinal (GI) and genitourinary (GU) effects. The median age of the patients was 75 (range: 56-85) years. The median follow-up was 14 (range: 0-32) months. The treatment was well tolerated by the majority of patients, with all completing the planned treatment without interruptions. No acute or late GU and GI toxicities ≥ G3 were observed. For acute GU toxicity, 18 (42%) patients reported symptoms, but only two (5%) experienced G2 toxicity. For acute GI toxicity, 12 (28%) patients presented symptoms, with only one (2%) showing G2 toxicity. Currently, data on late toxicity are available for 40 patients. Nine (23%) patients experienced late GU toxicity, with only one (3%) having G2 toxicity. Eleven (28%) patients reported late GI toxicity, of which six (15%) were G2. All of these patients required only symptomatic topical therapy with clinical benefit, except for one who underwent endoscopic therapy with argon plasma coagulation. This work demonstrates that prostate SBRT using Radixact Tomotherapy with Synchrony is safe; however, it is necessary to expand the sample size and extend follow-up to evaluate late toxicity and clinical outcomes.

## Introduction

Radiotherapy plays a crucial role among the various therapeutic options with curative purpose for prostate cancer (PC). In particular, intensity-modulated radiation therapy (IMRT) represents the therapeutic standard, in association or not with androgen deprivation therapy (ADT), for patients with localized PC treated with external beam radiation therapy (EBRT) [1-4]. Recent advancements have shifted radiation regimens from conventional fractionation to moderate and ultra-hypofractionation [5-7]. Stereotactic body radiation therapy (SBRT), a highly conformal EBRT technique delivering a limited number of high-dose fractions to the target volume while minimizing dose to adjacent healthy tissues, is gaining significant interest for localized PC, due to its unique radiobiological characteristic, i.e., the relatively slow proliferation, characterized by a low α/β ratio (approximately 1.5 compared to neighboring healthy tissues), potentially leading to improved tumor control with higher doses per fraction without increased toxicity and with the benefit of a shorter overall treatment duration [8-10]. The prostate's location in a dynamic anatomical region, subject to motion from variable rectal and bladder filling and bowel movement, poses a challenge to radiotherapy accuracy [11-15]. Intrafraction prostate motion can compromise treatment delivery, making its management crucial. This study aims to evaluate the safety, specifically acute and late gastrointestinal (GI) and genitourinary (GU) toxicities, of SBRT in patients with localized PC using Radixact Tomotherapy with Synchrony fiducial tracking, a system designed to detect target movement and correct radiotherapy delivery.

## Materials and methods

This retrospective single-center study analyzed data from patients with a histological diagnosis of localized PC who underwent SBRT treatment. All patients provided informed consent for the use of personal data and treatment after receiving adequate information about the chosen radiotherapy and potential acute and chronic side effects. ADT prescription was assessed based on disease risk. For Synchrony treatment, three 0.40x10 mm gold cylindrical fiducials (Gold Anchor MR+) were intraprostatically implanted by a radiation oncologist with urologist assistance, following antibiotic and anti-inflammatory prophylaxis and bowel preparation (one enema the evening before and two hours prior). Implantation was performed under short-term local anesthesia with ultrasound guidance, and correct placement was verified with ultrasound imaging. Approximately 10 days post-implantation, after fiducial stabilization, a non-contrast-enhanced axial simulation computed tomography (CT) was acquired for treatment planning, following bowel preparation (enema the evening before and morning of operation), bladder voiding, and intake of 500 mL of water 30 minutes prior. A low-fiber diet was encouraged. This same preparation was requested by patients throughout the entire treatment period. Patients were simulated supine with a headrest and knee pillow supports. Simulation CT images were reconstructed with a 2 mm slice thickness. Magnetic resonance imaging (MRI) images were fused rigidly using MIM Software (MIM Software Inc., Cleveland, OH) for improved target and organ-at-risk (OAR) definition. The prostate was contoured as the clinical target volume (CTV), and a 4 mm margin was added in all directions to create the planning target volume (PTV), except posteriorly (3 mm). The following OARs were contoured: bladder, rectum, femoral heads, and penile bulb. Contours were exported to the treatment planning system (Precision® version 3.3.1.3, Accuray Inc., Sunnyvale, CA) for Tomotherapy. A total dose of 36.25 Gy was prescribed in five daily fractions of 7.25 Gy on alternate days. Required target coverage was at least 95% of the PTV receiving at least 95% of the prescribed dose (V95%>95%). Table 1 shows the dose-volume constraints for organs at risk.

**Table 1:** Dose-volume constraints for OARs OARs: organs at risk

OAR	Dose (Gy)	Maximum volume (% or cc) mandatory	Maximum volume (% or cc) optimal
	18.1	50%	
Rectum	29	20%	
	36	2 cc	1 cc
Bladder	18.1	40%	
	37	10 cc	5 cc
Femoral heads	14.5		5%
Penile bulb	29.5		50%

The Synchrony system offers several distinct methods to accurately account for patient and tumor motion: fiducial tracking without respiratory modeling, fiducial tracking with respiratory modeling, and lung tracking with respiratory modeling. The first, used to treat patients with PC, uses a model based on the motion of gold fiducials placed within the target using X-ray images. The Synchrony treatment plan was developed by localizing fiducials as tracking targets, selecting 3-6 radiographic angles based on target visibility on digitally reconstructed radiographs (DRRs). During treatment planning, a 2.5 cm jaw width was selected for all plans based on the target range of motion, target size, and treatment efficiency. This choice optimized homogeneity and conformity while maintaining reasonable treatment times. A simulation plan similar to the Synchrony plan but without the MV beam was generated for delivery simulation. For each patient, both the Synchrony plan and the non-Synchrony plan were created to be used in case the Synchrony plan could not be delivered.

Prior to simulation and each treatment, cone beam computed tomography (CBCT) was performed for daily image guidance to verify positioning and bladder/rectal filling. Approximately 15 days post-implantation, a delivery simulation session assessed model reliability.

Input parameters were provided to help define the model and the behavior of the system. Once produced, the model was regularly checked for accuracy as data was obtained from updated radiographs. The parameters were displayed to the user to provide feedback on tracking, and if user-specified thresholds were exceeded, the model was updated or the treatment was paused. Accuray provides input thresholds for the following parameters: target offset (20 mm), potential difference (30 mm), center of motion deviation (30 mm), rigid body (15 mm), and auto pause delay (30 seconds).

If the simulation was deemed valid, the first treatment session occurred the following day (with personalized anti-inflammatory support). Otherwise, the non-Synchrony plan was used.

For each fraction, pre-treatment X-rays were acquired at preselected angles to initiate target tracking and motion modeling. The beam was initiated while jaws and multileaf collimators (MLCs) tracked the target motion. Patient treatment was carefully observed for necessary interventions (pausing, acquiring additional images, and resuming treatment) as the motion model was updated with each new X-ray image.

Patients were clinically evaluated at baseline, during treatment, two weeks post-treatment, and then every three months. Acute and late GU and GI toxicity were evaluated using the Common Terminology Criteria for Adverse Events (CTCAE) version 5, with acute toxicity defined as events within the first 90 days post-treatment and late toxicity thereafter.

## Results

This work included data from 43 patients with localized PC who underwent SBRT between June 2022 and February 2025. The median age at diagnosis was 75 (range: 56-85) years. According to the National Comprehensive Cancer Network (NCCN) risk classification, nine (21%) patients were classified as low risk, 15 (35%) as favorable intermediate risk, 13 (30%) as unfavorable intermediate risk, and six (14%) as high risk. Fourteen (33%) patients received ADT. Treatment was delivered in five fractions for a total dose of 36.25 Gy on alternate days. The median follow-up duration was 14 (range: 0-32) months. A summary of the clinical characteristics of the patients is reported in Table 2.

**Table 2:** Clinical characteristics of patients NCCN: National Comprehensive Cancer Network

Characteristics	Number	%
Age at diagnosis		
Median, range	75 (56-85)	
Gleason score		
6 (3+3)	14	33%
7 (3+4)	18	42%
7 (4+3)	7	16%
8 (4+4)	3	7%
9 (4+5)	1	2%
NCCN risk group		
Low	9	21%
Favorable intermediate	15	35%
Unfavorable intermediate	13	30%
High	6	14%
Androgen deprivation therapy		
Yes	14	33%
No	29	67%
Follow-up		
Median, range	14 (0-32)	

Treatment was well tolerated by the majority of patients. No patients experienced grade ≥ 3 GI or GU toxicity, either acutely or late. All patients completed the planned treatment without any interruptions. A summary of the toxicities found is present in Table 3.

**Table 3:** Acute and late GU and GI toxicity according to CTCAE version 5 GU: genitourinary, GI: gastrointestinal, CTCAE: Common Terminology Criteria for Adverse Events

Toxicity	G0	G1	G2	G3	G4	G5
Acute GU, number (%)	25 (58%)	16 (37%)	2 (5%)	0 (-)	0 (-)	0 (-)
Acute GI, number (%)	31 (72%)	11 (26%)	1 (2%)	0 (-)	0 (-)	0 (-)
Late GU, number (%)	31 (77%)	8 (20%)	1 (3%)	0 (-)	0 (-)	0 (-)
Late GI, number (%)	29 (72%)	5 (13%)	6 (15%)	0 (-)	0 (-)	0 (-)

Regarding acute GU toxicity, 18 (42%) patients reported symptoms, with only two (5%) patients experiencing G2 toxicity. For acute GI toxicity, 12 (28%) patients presented with symptoms, and similarly, only one (2%) patient experienced G2 toxicity.

Late toxicity data are currently available for 40 patients. Nine (23%) patients experienced late GU toxicity, with only one (3%) patient developing G2 toxicity. Eleven (28%) patients reported late GI toxicity, of whom six (15%) experienced G2 toxicity. In this context, it is important to note that all of these patients required only symptomatic topical therapy with clinical benefit, except for one patient who underwent endoscopic therapy with argon plasma coagulation.

## Discussion

Our retrospective analysis of 43 localized PC patients treated with SBRT using Radixact Tomotherapy with Synchrony fiducial tracking demonstrates a favorable safety profile with short- to medium-term follow-up. All patients completed the planned ultra-hypofractionated regimen of 36.25 Gy in five fractions without treatment interruptions. Notably, we observed no acute or late GU and GI toxicities of G3 or higher. While acute G2 GU and GI toxicity rates were 5% and 2%, respectively, and late G2 toxicities were observed in 3% for GU and 15% for GI, with the latter primarily managed with symptomatic topical therapy, except for one case requiring endoscopic intervention.

It is interesting to compare these observations with the emerging data from randomized phase 3 trials and with the clinical experience in the implementation of advanced radiotherapy techniques.

The HYPO-RT-PC trial compared an ultra-hypofractionated radiotherapy regimen (42.7 Gy in seven fractions, three days per week for 2.5 weeks) with a conventional regimen (78 Gy in 39 fractions, five days per week for eight weeks) and reported five-year outcomes [16]. Although our SBRT regimen (36.25 Gy in five fractions) differs in total dose and fractionation, the results of HYPO-RT-PC support the efficacy and tolerability of shorter fractionation regimens compared to conventional radiotherapy. Widmark et al.'s data demonstrated non-inferiority in terms of biochemical failure and comparable late toxicity between the two arms [16]. Our study, with a shorter median follow-up, suggests a low incidence of toxicity, which is encouraging in the context of even more hypofractionated regimens such as SBRT.

The non-inferiority phase 3 randomized PACE-B trial suggests that SBRT (36.25 Gy in five fractions over 1.5 weeks) does not increase acute toxicity, both GU and GI, at 12 weeks compared to conventional/hypofractionated regimens (78 Gy in 39 fractions over 7.8 weeks/62 Gy in 20 fractions over four weeks) [17]. In October 2022, at two years of follow-up, the same authors confirmed that this treatment is safe and associated with low rates of side effects [18].

Chen et al.'s experience on the clinical implementation of motion tracking with helical Tomotherapy provides an important technical context for our work [19]. They described that the integration of tracking with jaws and multileaf collimator during Tomotherapy delivery supports the feasibility and potential clinical benefits of the technique we used. The ability to correct prostate motion in real time, made possible by the Synchrony system and the implanted fiducials, is crucial for ensuring optimal target coverage and minimizing the irradiation of surrounding healthy tissues, potentially contributing to the low toxicity rates observed in our study.

The study by Grün et al. on the geometry and detection rates of prostate fiducials underscores the importance of effective marker implantation for accurate guidance during radiotherapy [20].

The use of three gold fiducials in our study, implanted under ultrasound guidance, is consistent with recommendations for reliable tracking. The clear visualization of the fiducials before and during treatment was fundamental for the effective implementation of the Synchrony system and the precision of dose delivery. Grün et al.'s findings on the reliability of fiducial detection support the validity of our approach in ensuring high-precision image-guided treatment [20].

Our initial safety outcomes align with the findings presented in the case report by Shintani et al., which described a successful treatment of a PC patient using Tomotherapy with Synchrony fiducial tracking for SBRT [21]. While their report focused on a single case, the absence of significant acute toxicity supports the feasibility and initial safety of this approach. Our larger cohort provides further evidence that the real-time motion tracking and correction capabilities of the Synchrony system may contribute to minimizing treatment-related side effects by ensuring accurate dose delivery despite intrafraction prostate motion.

The dosimetric comparison by Manabe et al. examined SBRT plans using Tomotherapy and CyberKnife in patients with a hydrogel spacer [22]. Being a planning comparison, patients did not undergo fiducial insertion and were treated with IMRT using Tomotherapy. Although their dosimetric study did not report clinical outcomes, the reasonable profile demonstrated by Tomotherapy for prostate SBRT suggests the potential for effective and safe treatment, a finding supported by our initial clinical results, which add clinical evidence to their dosimetric potential.

Furthermore, the dosimetric comparison between CyberKnife and the Radixact system with Synchrony by Kong et al. for both lung cancer and PC provides a broader context for understanding the capabilities of the Radixact platform with motion tracking [23]. While their study focused on dosimetric parameters, the ability of both systems to deliver precise SBRT with motion management suggests that the low severe toxicity observed in our study might be attributable to the inherent precision offered by these technologies.

However, it is important to acknowledge the limitations of our study, including its retrospective nature, relatively small sample size, and the median follow-up of 14 months, which might not be sufficient to fully capture the spectrum of late toxicities, although previous experiences report a substantial tendency for late toxicity to remain stable after one year of follow-up [24]. While the late G2 GI toxicity rate of 15% warrants attention and ongoing monitoring, the fact that most cases were manageable with topical therapy is encouraging.

## Conclusions

Our initial findings suggest that prostate SBRT using Radixact Tomotherapy with Synchrony fiducial tracking is a safe treatment approach with acceptable acute and early late toxicity rates. These results are consistent with the emerging evidence from other studies utilizing similar technologies. However, as we acknowledge, expanding the patient cohort and extending the follow-up period are crucial to definitively assess the long-term safety profile and clinical outcomes of this treatment modality for localized PC. Future research should also focus on identifying potential risk factors for late toxicities to further optimize patient selection and treatment strategies.
